# What Is a Clinical Lecture?

**Published:** 1852-12

**Authors:** 


*What is a Clinical Lecture?*—The editor of the New York Medical Times answers this question by the following definition quoted from the Edinburgh Monthly Journal: "Judging from those that are perpetually appearing in print, it is sometimes a tritical medical essay, not necessarily delivered, and unnecessarily printed."

